# Deconstruct the link between gut microbiota and neurological diseases: application of Mendelian randomization analysis

**DOI:** 10.3389/fcimb.2025.1433131

**Published:** 2025-03-06

**Authors:** Jingqiu Li, Xinyang Hu, Xinyu Tao, Yuming Li, Wan Jiang, Mingtao Zhao, Zhehui Ma, Bangjie Chen, Shuyan Sheng, Jiaye Tong, Haibo Zhang, Bing Shen, Xiaomei Gao

**Affiliations:** ^1^ Second Clinical Medical College, Anhui Medical University, Hefei, China; ^2^ Frist Clinical Medical College, Anhui Medical University, Hefei, China; ^3^ Department of Neurology, Affiliated Drum Tower Hospital, Medical School of Nanjing University, Nanjing, China; ^4^ Dr. Neher’s Biophysics Laboratory for Innovative Drug Discovery, State Key Laboratory of Quality Research in Chinese Medicine, Macau University of Science and Technology, Macao, Macao SAR, China

**Keywords:** Mendelian randomization, gut flora, neurological disorders, single nucleotide polymorphisms, inflammation, genetics

## Abstract

**Background:**

Recent research on the gut-brain axis has deepened our understanding of the correlation between gut bacteria and the neurological system. The inflammatory response triggered by gut microbiota may be associated with neurodegenerative diseases. Additionally, the impact of gut microbiota on emotional state, known as the “Gut-mood” relationship, could play a role in depression and anxiety disorders.

**Results:**

This review summarizes recent data on the role of gut-brain axis in the pathophysiology of neuropsychiatric and neurological disorders including epilepsy, schizophrenia, Alzheimer’s disease, brain cancer, Parkinson’s disease, bipolar disorder and stroke. Also, we conducted a Mendelian randomization study on seven neurological disorders (Epilepsy, schizophrenia, Alzheimer’s disease, brain cancer, Parkinson’s disease, bipolar disorder and stroke). MR-Egger and MR-PRESSO tests confirmed the robustness of analysis against horizontal pleiotropy.

**Conclusions:**

By comparing the protective and risk factors for neurological disorders found in our research and other researches, we can furtherly determine valuable indicators for disease evolution tracking and potential treatment targets. Future research should explore extensive microbiome genome-wide association study datasets using metagenomics sequencing techniques to deepen our understanding of connections and causality between neurological disorders.

## 1 Introduction

The fields of neurology and microbiology have followed separate paths of development, with little convergence, save in instances involving bacterial or viral diseases of the central nervous system, prion infections, Guillain-Barré syndrome, and septic or hepatic encephalopathies. In the last twenty years, a significant biological advancement has taken place, recognizing the involvement of the gut microbiota and microbiome in maintaining homeostasis and regulating several essential body systems, including the central nervous system (CNS) (Cryan et al., 2020). Emerging evidence indicates that the gut microbiota play a crucial role in the bidirectional communication between the gut and the brain suggesting that the gut microbes may shape neural development, modulate neurotransmission and affect behavior, and thereby contribute to the pathogenesis and/or progression of many neurodevelopmental, neuropsychiatric, and neurological conditions. There are Neurological disorders contribute significantly to the physical and economic burden experienced by individuals due to their propensity to manifest as chronic and enduring conditions. While there has been a decline in age-standardized morbidity, mortality, and morbidity rates related to neurological illnesses in certain countries, it is important to note that the overall worldwide prevalence of persons affected by neurological disorders, as well as cases of murder and disability, has grown over the last 25 years (Lobo et al., 2000; Feigin et al., 2016; Tysnes and Storstein, 2017). At the same time, in recent years, the mechanism of neurological disorder has been continuously explored, including the activation of PUFA-associated neuroinflammation by gut microbiota (Chen et al., 2022). The identification of these mechanisms has enabled researchers to develop more precise therapeutic and preventive strategies for neurological illnesses. However, the precise cause of the illness remains unidentified.

The gut microbiota (GM) has been associated with the pathogenesis of inflammatory, metabolic, psychiatric, and immunological disorders, as well as the regulation of neurotransmitter activity (Patterson et al., 2016; Strandwitz, 2018; Schoeler and Caesar, 2019). There has been a significant increase of foundational research suggesting that the microbiome plays a crucial role in the proper development and upkeep of the brain. The body of evidence derived from clinical and animal studies pertaining to the correlation between the microbiome and neurological disorders is steadily expanding. The significance of the microbiota is strongly supported by convincing data in several medical conditions, including Parkinson’s disease (Sampson et al., 2016), multiple sclerosis (Correale et al., 2022), and autistic spectrum disorder (Sharon et al., 2019). Furthermore, there is an emerging awareness of its relevance in Alzheimer’s disease (Chen et al., 2022) and stroke (Spychala et al., 2018). There is evidence that the gut microbiota communicates with the brain through Vagus nerve (Forsythe et al., 2014). Several studies have shown a strong correlation between gastrointestinal disorders, anxiety, depressive symptoms, and even personality differences (Tosic-Golubovic et al., 2010). Another study showed that children with (or without) gastrointestinal problems were more likely to show an increase in symptoms of irritability, social withdrawal and anxiety (Nikolov et al., 2009). Similar findings were found in children with ASD (Mazefsky et al., 2014). Thus, these findings summarize that the intrinsic systems involved in neurochemical transmission and neuronal development are indeed affected by changes in gut microbial diversity (Ben-Azu et al., 2023).Nonetheless, it is essential to use prudence in drawing conclusions from the available data, since the current stage of research is in its nascent phase. A significant proportion of the studies exhibit inadequate statistical power, as well as biases in participant selection, variations in sampling and sequencing methods, inconsistencies in bioinformatics pipelines, statistical methodologies, and the presence of confounding factors. Therefore, it is necessary to conduct more research that is both well-controlled and well-designed in order to get a comprehensive understanding of the intricate processes behind the involvement of the gut-brain axis in neurological illnesses. In this review, we aimed to summarize data on the role of the gut-brain axis in the pathogenesis of neuropsychiatric and neurological diseases, namely epilepsy, schizophrenia, Alzheimer’s disease, brain cancer, Parkinson’s disease, bipolar disorder and stroke, in order to provide a current framework in this rapidly evolving research area and deepen our understanding of connections and causality between neurological disorders.

Mendelian randomization (MR) is a research tool that use genetic variation to investigate the causal impact of functions or phenotypes on illness outcomes, akin to randomized controlled trials (RCTs) (Manousaki et al., 2021). On the other hand, the approach referred to as MR employs instrumental variables (IVs) to address possible confounding factors, rather than addressing them as distinct treatments. In the presence of a causal relationship between an exposure and an outcome, it is possible to estimate the causal effect of the exposure on the outcome using instrumental variables. This is feasible when the instrumental variable is associated with the exposure but not influenced by any confounding factors that affect the relationship between the exposure and the outcome. Additionally, there should be no direct causal link from the instrumental variable to the outcome, except through the exposure. This estimation can be done using either a single instrumental variable or a set of instrumental variables for the exposure. MR has been widely used across several industries and has yielded substantial outcomes (Emdin et al., 2017). In our study, we used GM taxa as the independent variables and neurological illnesses as the dependent variables in order to conduct a Mendelian randomization analysis. The main purpose of our review was to investigate the potential causal relationship between GM and neurological diseases, and to compare the results with those of MR Studies conducted by others to find differences and explore the potential association. In this study, using the genome-wide association study (GWAS) summary statistics from the MiBioGen and FinnGen consortiums, whose aims to provide an important foundation for precision medicine and drug development by in-depth exploration of genetic variants associated with disease by integrating large-scale genomic data and phenotypic information, a two-sample MR analysis was conducted to evaluate the causal association between gut microbiota and Neurological disorders.

## 2 Materials and methods

### 2.1 The assumptions and study design of MR

This study employed a two-sample Mendelian randomization (MR) analysis to assess the causal association between genetic microbiota (GM) taxa and various neurological disorders. The analysis utilized publicly available summary-level data from genome-wide association studies (GWASs) for both the GM taxa exposures and the neurological disorder outcomes, including Epilepsy, Schizophrenia, Alzheimer’s disease, Brain cancer, Parkinson’s disease, bipolar disorder, and Stroke. In order to ensure the integrity of the MR analysis, it is essential that three assumptions be satisfied on each occasion. (1) It is imperative that the genetic variants utilized in the analysis exhibit a substantial correlation with the exposure under investigation. (2) The genetic variants chosen as instrumental variables (IVs) for the exposure must be independent of any confounding factors that are associated with both the exposure and the outcome. (3) The presence of horizontal pleiotropy, wherein the IVs can solely influence neurological disorders via GM taxa, should be absent in the study (Wu et al., 2020).

### 2.2 Ethics statement

The present research used publicly available, de-identified summary-level data that may be accessed without cost, in order to examine the association between taxa of GM and neurological diseases. All the GWAS studies used in this study received approval from the institutional ethics committees to ensure compliance with ethical guidelines.

### 2.3 Exposure sources of GM taxa

Kurilshikov et al. (2020) used data obtained from the MiBioGen collaboration in order to examine the association between gut microbiota (GM) and genetic variation, as reported in their publication with the PubMed (Kurilshikov et al., 2021). The dataset included of profiles of 16S rRNA gene sequencing and genotyping information obtained from a total of 18,340 people of European descent. These individuals were recruited from 25 different cohorts located in 11 countries. Based on the provided data, the research team successfully discovered a total of 122,110 variant sites across 211 taxa, ranging from the genus to the phylum level. The IVs representing microbial species at five taxonomic levels were found from the Genome-Wide Association Study (GWAS) conducted by the MiBioGen collaboration. Additional details on the GM data included in this research may be obtained from the original article.

To adhere to the three fundamental principles of Mendelian randomization (MR) and assure the precision of the findings, a comprehensive quality assessment was conducted on all single nucleotide polymorphisms (SNPs). In order to ensure the statistical significance of the chosen SNPs in relation to the exposure, all SNPs related with the gut microbiota taxa achieved a genome-wide significance criterion of P < 5×10^-8^. Furthermore, in order to ensure a thorough and comprehensive conclusion, a distinct set of SNPs that fell below the significance threshold of the entire locus (1× 10^-5^) were chosen as instrumental variables. Additionally, a linkage disequilibrium (LD) analysis was conducted, with a threshold of R^2^ < 0.001 and a clumping distance of 10,000kb, to satisfy the assumptions of Mendelian randomization. To mitigate the potential impact of alleles on the causal association between genetically modified taxa and neurological disorders, palindrome SNPs were excluded from the analysis.

To address the probable presence of weak instrumental bias, the strength of the instrumental variable was assessed by means of the F statistic. The F statistic was computed using the formula F = R^2^×(N-2)/(1-R^2^), where N represents the sample size. If the F statistic is more than 10 (Feng et al., 2022), it may be concluded that the correlation between the independent variables and exposure is strong enough to protect the findings of the MR study from being influenced by weak instrumental bias.

### 2.4 Outcome source of neurological disorders

The summary-level data for seven neurological disorders were extracted from a large-scale mate-analysis GWAS from the FinnGen biobank. The ID of the epilepsy data is finn-b-G6_EPLEPSY. This GWAS whose number of SNPs is 16,380,349 included 182,367 European adult female and male subjects and consisted of 6,260 cases and 176,107controls. The Schizophrenia data with the ID number ieu-b-5099 came from a 2022 study(35396580). The study identified biological processes associated with Pathophysiology in schizophrenia, showed convergence in the association of Common and rare variants in schizophrenia and neurodevelopmental disorders (Trubetskoy et al., 2022). And provide the resources of priority genes and variants to advance the mechanism research. The GWAS ID of the remaining five neurological disorders are shown in detail in the follow-up results.

### 2.5 Statistical analysis

**Figure 1:**
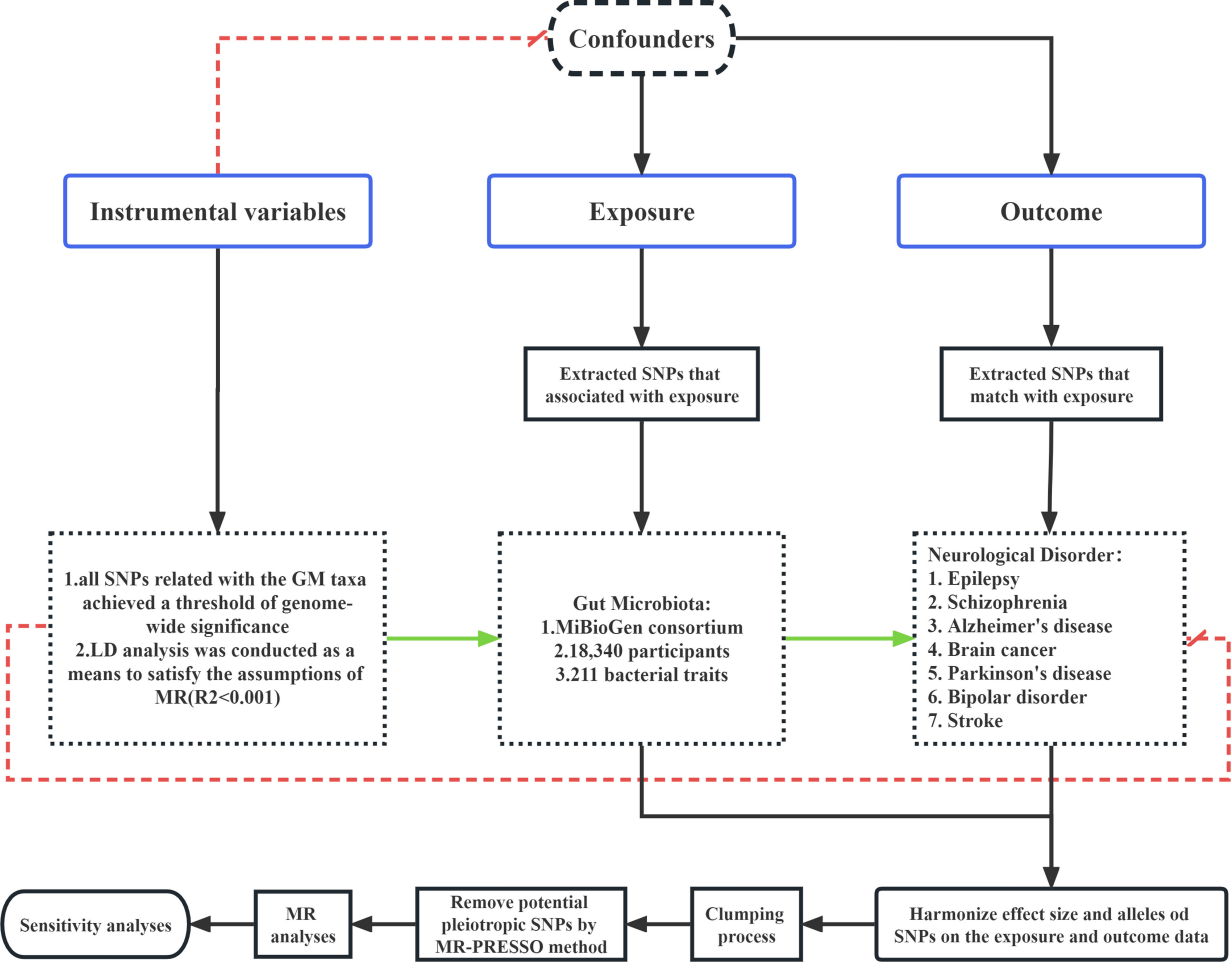
Summary of the main assumptions and methods used in MR analysis.

In this research, all statistical analyses were performed using the R software (Version 4.1.1). The MR analysis was conducted using the “TwoSampleMR” package in the R to examine the possible causal association between GM taxa and neurological disorders. A significance threshold of P < 0.05 was used to indicate the presence of probable causal influence, based on statistical analysis. The provided visual representation of process, labelled as **Figure 1**, is presented for reference and analysis.

#### 2.5.1 MR estimates

A range of methodologies, including inverse variance weighting (IVW), weighted mean, weighted median (WM), and the MR-Egger test, are used to establish the credibility and soundness of the research. In order to provide comprehensive estimations about the influence of GM on neurological disorders, the IVW approach is used. This technique aggregates Wald values for each single nucleotide polymorphism (SNP) via the utilization of a meta-analysis methodology. The choice between the fixed or random effects model for the IVW test was determined based on the existence or absence of heterogeneity. We used a random-effect IVW model when significant heterogeneity (P < 0.05) was seen in the given conditions. In addition to our primary analyses, we conducted supplementary analyses using the weighted median (WM) approach and the Mendelian randomization-Egger (MR-Egger) test. In instances where the fraction of SNPs exhibiting variability over 50%, we considered the results obtained from the WM analysis to suggest robust causal effects. The reliability of the MR-Egger findings was considered satisfactory when the fraction of pleiotropic single nucleotide polymorphisms exceeded 50%. However, it is important to note that MR-Egger calculations might be inaccurate and greatly influenced by genetic variants that deviate significantly from the norm. The statistical analyses were conducted using the R program (Version 4.1.1), and a significance level of P < 0.05 was used as the threshold for statistical significance.

#### 2.5.2 Sensitivity analysis

In the present work, we used the MR-Egger and MR-PRESSO regression techniques to assess the probable existence of pleiotropy in the single nucleotide polymorphisms (SNPs) utilized as instrumental variables (IVs). Horizontal pleiotropy was deemed to be missing if the p-value (P) exceeded 0.05. The assessment of heterogeneity was conducted using Cochrane’s Q test, and IVs with a significance level of P < 0.05 were considered to exhibit heterogeneity. In addition, we conducted a sensitivity analysis referred to as “leave-one-out” in the Mendelian randomization (MR) methodology. This analysis included systematically excluding each single nucleotide polymorphism (SNP) to assess its possible influence on the results.

## 3 Results

### 3.1 Selection of IVs related to GM

Following quality control measures including LD effects and palindromic analysis, we found SNPs to be IVs associated with 211 taxa for neurological disorder we’ve been working on (with threshold of *P* < 1×10^-5^). The IVs we are looking for are different for each neurological disorder, so we show the number of all IVs in a diverse set of taxa (**Table 1**).

**Table 1:** Selection of IVs after quality control.

Epliepsy			Schizophrenia			Alzheimer's disease			Brain Cancer		
Taxonomies	Taxa	Ivs	Taxonomies	Taxa	Ivs	Taxonomies	Taxa	Ivs	Taxonomies	Taxa	Ivs
phylum	9	52	phylum	9	52	phylum	9	54	phylum	9	52
class	16	101	class	16	101	class	16	100	class	16	101
order	20	124	order	20	121	order	20	121	order	20	121
family	35	212	family	35	210	family	35	207	family	35	210
genus	131	734	genus	131	724	genus	131	712	genus	131	725
total	211	1223	total	211	1208	total	211	1194	total	211	1209
Parkinson's diease			Bipolar Disorder			Stroke					
Taxonomies	Taxa	Ivs	Taxonomies	Taxa	Ivs	Taxonomies	Taxa	Ivs			
phylum	9	52	phylum	9	53	phylum	9	54			
class	16	101	class	16	102	class	16	102			
order	20	124	order	20	122	order	20	124			
family	35	212	family	35	209	family	35	218			
genus	131	733	genus	131	724	genus	131	741			
total	211	1222	total	211	1210	total	211	1239			

Additionally, SNPs with a significance threshold of *P* < 5×10^-8^ were identified as IVs that were associated with 211 bacterial taxa for neurological disorders. Meanwhile, not much SNPs passed quality control measures and were found suitable to be utilized as IVs when considering the GM as a whole (with threshold of *P* < 1×10^-5^). Their details are shown in **Supplementary Materials** (**Supplementary Table 1-1**-**Supplementary Table 7-1**-**Supplementary Table 1-2**-**Supplementary Table 7-2**).

### 3.2 Results of MR analysis

**Figure 2:**
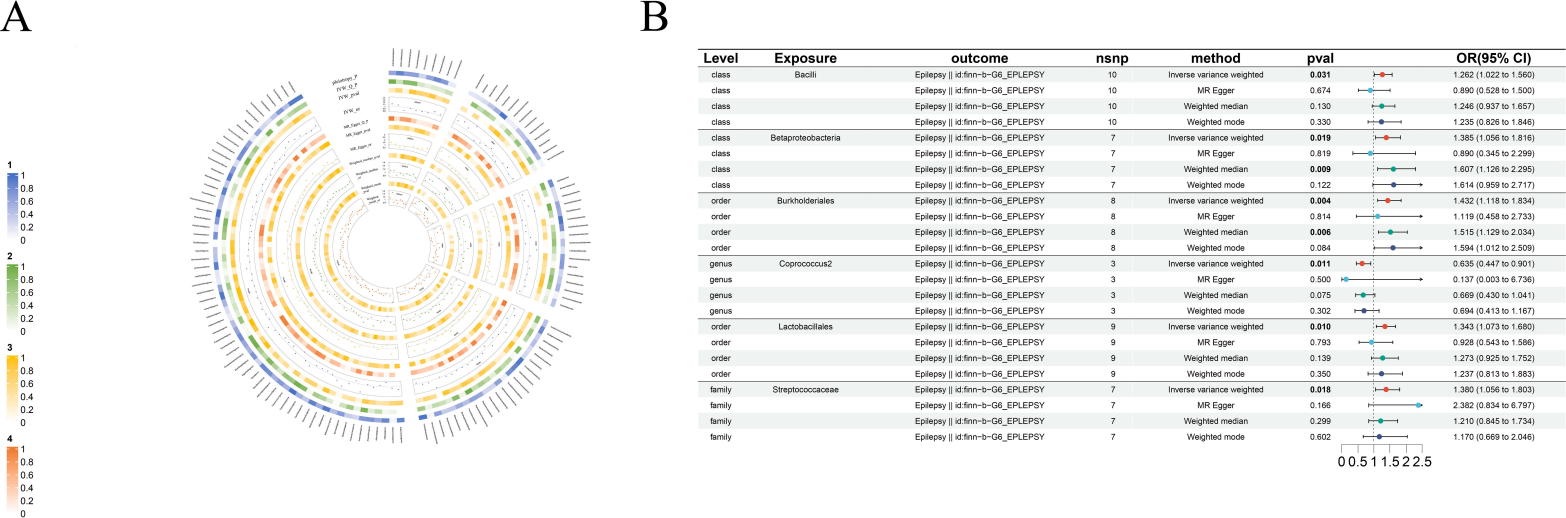
Causal analysis of GM on Epilepsy. **(A)** All results of MR analysis and sensitivity analysis between GM and Epilepsy; **(B)** MR results of GM taxa with a causal relationship to Epilepsy.

**Figure 3:**
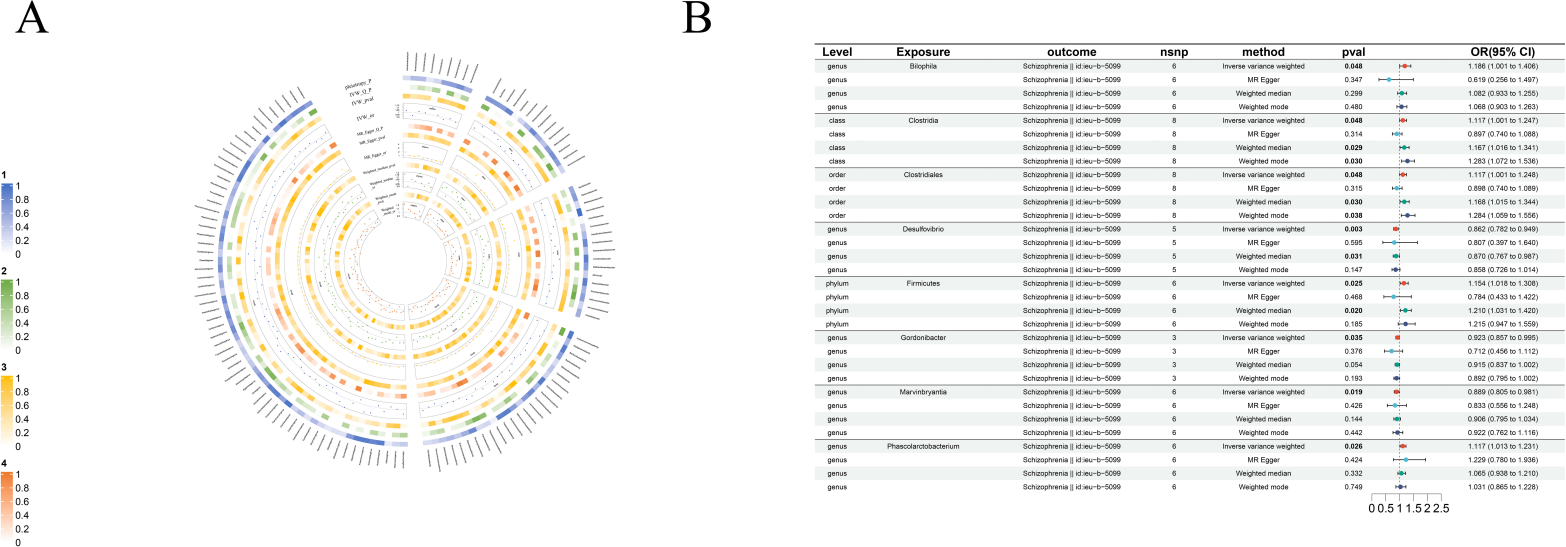
Causal analysis of GM on Schizophrenia. **(A)** All results of MR analysis and sensitivity analysis between GM and Schizophrenia; **(B)** MR results of GM taxa with a causal relationship to Schizophrenia.

**Figure 4:**
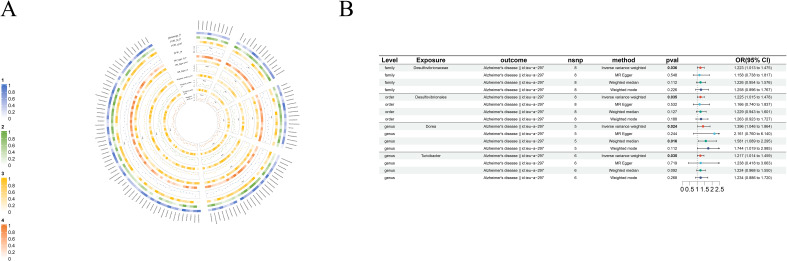
Causal analysis of GM on Alzheimer’s disease. **(A)** All results of MR analysis and sensitivity analysis between GM and Alzheimer’s disease; **(B)** MR results of GM taxa with a causal relationship to Alzheimer’s disease.

**Figure 5:**
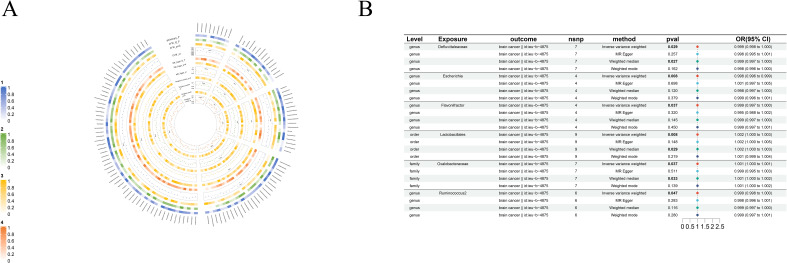
Causal analysis of GM on Brain cancer. **(A)** All results of MR analysis and sensitivity analysis between GM and Brain cancer; **(B)** MR results of GM taxa with a causal relationship to Brain cancer.

**Figure 6:**
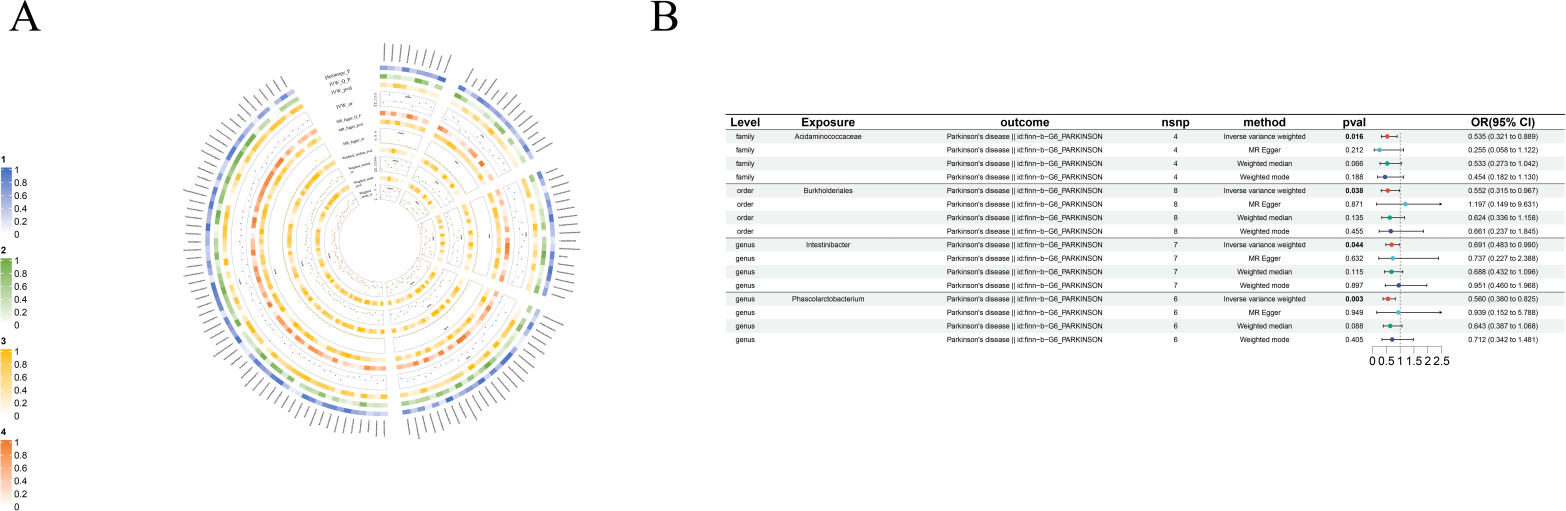
Causal analysis of GM on Parkinson’s disease. **(A)** All results of MR analysis and sensitivity analysis between GM and Parkinson’s disease; **(B)** MR results of GM taxa with a causal relationship to Parkinson’s disease.

**Figure 7:**
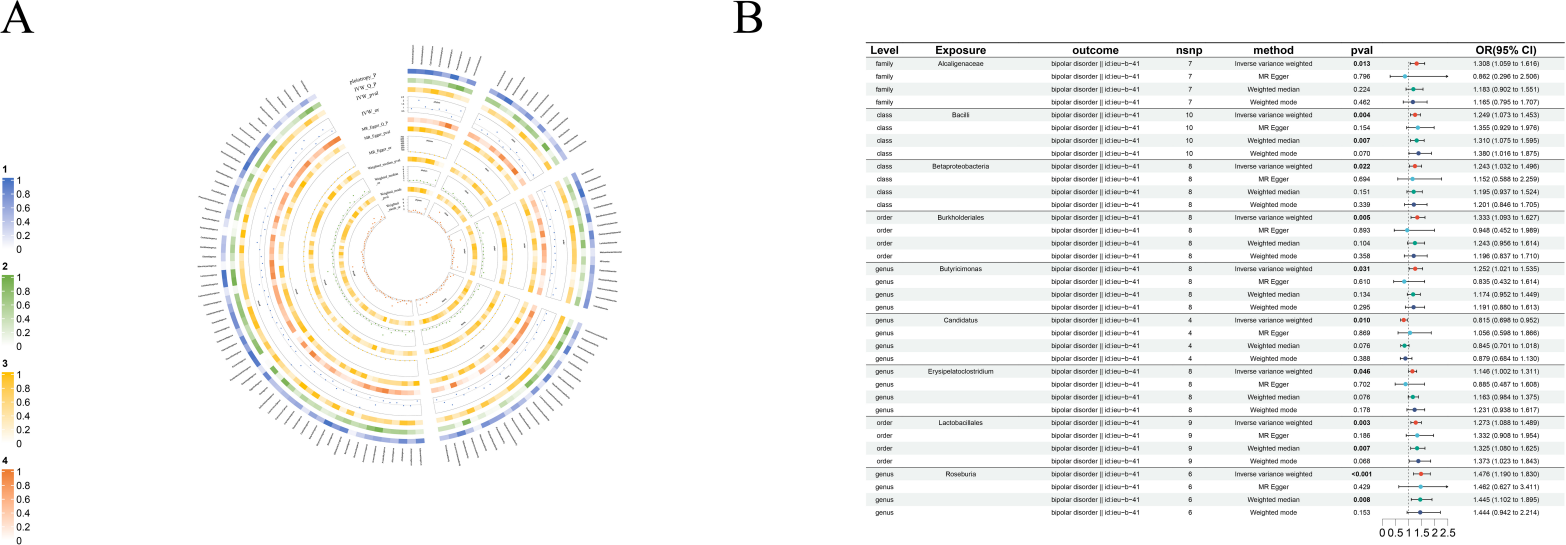
Causal analysis of GM on Bipolar disorder. **(A)** All results of MR analysis and sensitivity analysis between GM and Bipolar disorder; **(B)** MR results of GM taxa with a causal relationship to Bipolar disorder.

**Figure 8:**
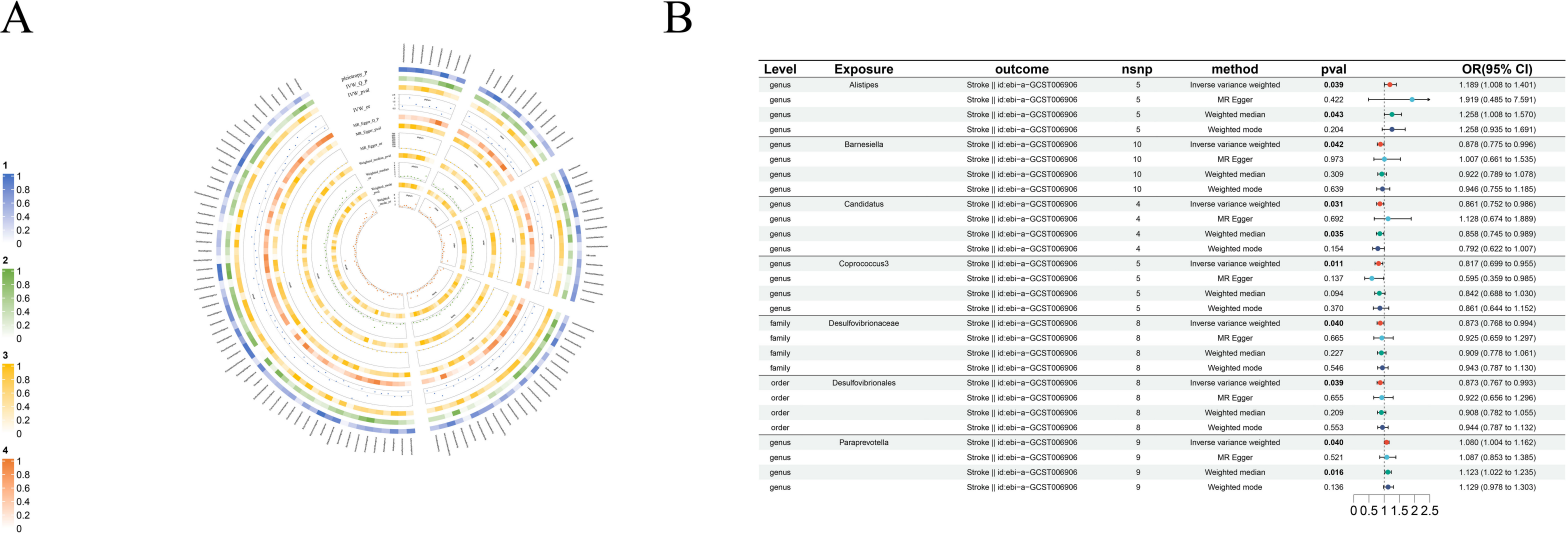
Causal analysis of GM on Stroke. **(A)** All results of MR analysis and sensitivity analysis between GM and Stroke; **(B)** MR results of GM taxa with a causal relationship to Stroke.

As part of the MR analysis, we observed a genetically predicted relative abundance of 87 different taxa. Furthermore, **Figures 2A**-**8A** provides a visual representation of the relationship between 211 bacterial taxa and neurological disorders. In **Figures 2B**-**8B**, information on the bacterial taxa closely associated with neurological disorders is presented. Other results are detailed as **Supplementary Table 1–3**-**Supplementary Table 7–3**. **Table 2** also shows how the gut microbiota as a whole affects a range of neurological disorders though GM as a whole only can be seen as a risk factor for Parkinson’s disease(p<0.05). **Supplementary Table 1-4**-**Supplementary Table 7-4** details the major information.

**Table 2:** MR results between GM and neurological disorders (P<5x10^-8^).

GM	Outcome	Method	Ivs	OR	95%CI	P	Q	Q-P	Intercept	P
Total	Epilepsy	Inverse variance weighted	15	0.897146	0.77-1.05	0.179114	25.329743	0.0314483	0.0114801	0.6230809
Total	Epilepsy	MR Egger	15	0.806463	0.52-1.26	0.361391				
Total	Epilepsy	Weighted median	15	0.803743	0.66-0.97	0.026169				
Total	Epilepsy	Weighted mode	15	0.733654	0.52-1.03	0.093077	24.845361	0.0241873		
Total	Schizophrenia	Inverse variance weighted	15	0.98977	0.92-1.07	0.794949	34.79479	0.001576	-0.01585	0.145174
Total	Schizophrenia	MR Egger	15	1.151684	0.94-1.41	0.200742				
Total	Schizophrenia	Weighted median	15	1.004571	0.93-1.08	0.903993				
Total	Schizophrenia	Weighted mode	15	1.000122	0.91-1.10	0.998049	29.3684	0.005802		
Total	Alzheimer's disease	Inverse variance weighted	13	0.969316	0.87-1.08	0.571839	12.104148	0.4373521	-0.011579	0.463315
Total	Alzheimer's disease	MR Egger	13	1.083177	0.80-1.47	0.619918				
Total	Alzheimer's disease	Weighted median	13	1.011571	0.86-1.18	0.885888				
Total	Alzheimer's disease	Weighted mode	13	1.094764	0.87-1.37	0.450538	11.500496	0.4023342		
Total	brain cancer	Inverse variance weighted	15	1.000657		0.061499	18.4598	0.186634	-1.46E-05	0.8876835
Total	brain cancer	MR Egger	15	1.000797		0.456611				
Total	brain cancer	Weighted median	15	1.000439		0.338584				
Total	brain cancer	Weighted mode	15	0.999909		0.906415	18.430392	0.1418508		
Total	Parkinson's disease	Inverse variance weighted	15	1.271756	1.04-1.55	0.017396	11.970951	0.6086357	-0.01545	0.58736
Total	Parkinson's disease	MR Egger	15	1.467358	0.85-2.52	0.188456				
Total	Parkinson's disease	Weighted median	15	1.32102	1.00-1.75	0.052012				
Total	Parkinson's disease	Weighted mode	15	1.565339	1.00-2.44	0.068528	11.66132	0.555591		
Total	bipolar disorder	Inverse variance weighted	15	1.072684	0.98-1.17	0.116992	13.608182	0.4792907	-0.011877	0.3534434
Total	bipolar disorder	MR Egger	15	1.201332	0.94-1.53	0.168933				
Total	bipolar disorder	Weighted median	15	1.085287	0.97-1.22	0.160502				
Total	bipolar disorder	Weighted mode	15	1.093227	0.95-1.26	0.249951	12.682104	0.4726558		
Total	Stroke	Inverse variance weighted	15	1.004007	0.94-1.08	0.910974	18.654921	0.1785584	0.0004087	0.9686976
Total	Stroke	MR Egger	15	1.00012	0.82-1.23	0.999097				
Total	Stroke	Weighted median	15	1.032908	0.94-1.13	0.491684				
Total	Stroke	Weighted mode	15	1.024565	0.91-1.28	0.696145	18.652624	0.1342742		

#### 3.2.1 Epilepsy

IVW shows *genus-Coprococcus2*(beta=-0.45, se=0.18, p-value=1,09×10^-2^, or=0.63, 95%CL,0.45-0.90) is protective factor for epilepsy and *class-Bcilli* (beta=0.23, se=0.11, p-value=3.09×10^-2^, or=1.26, 95%CL, 1.02-1.56), *class-Betaproteobacteria* (beta=0.33, se=0.14, p-value=1.85×10^-2^, or=1.38, 95%CL, 1.06-1.82), *order-Burkholderiales* (beta=0.36, se=0.13, p-value=4.44×10^-3^, or=1.43, 95%CL, 1.12-1.83), *order-Lactobacillales* (beta=0.29, se=0.11, p-value=9.96×10^-3^, or=1.34, 95%CL, 1.07-1.68) as well as *family-Streptococcaceae* (beta=0.32, se=0.14, p-value=1.85×10-2, or=1.38, 95%CL, 1.06-1.80) are risk factors for epilepsy. Even more remarkable, MR Egger shows there is a causal relationship between *genus-Oscillibacter* and epilepsy (**Figure 2B**).

#### 3.2.2 Schizophrenia

We found eight strong causal associations with schizophrenia: on one hand, *genus-Desulfovibrio* (beta=-0.15, se=0.05, p-value=2.63×10^-3^, or=0.86, 95%CL, 0.78-0.94), *genus-Gordonibacter* (beta=-0.08, se=0.04, p-value=3.55×10^-2^, or=0.92, 95%CL, 0.86-0.99) and *genus-Marvinbryantia* (beta=-0.12, se=0.05, p-value=1.88×10^-2^, or=0.89, 95%CL, 0.81-0.98) are protective factors for schizophrenia, on the other hand, the risk factors are *phylum-Firmicutes* (beta=0.14, se=0.06, p-value=2.54×10^-2^, or=1.15, 95%CL, 1.02-1.31), *class-Clostridia* (beta=0.11, se=0.06, p-value=4.85×10^-2^, or=1.12, 95%CL, 1.00-1.25), *order-Clostridiales* (beta=0.17, se=0.09, p-value=4.82×10^-2^, or=1.19, 95%CL, 1.00-1.25), *genus- Bilophila*(beta=0.23, se=0.11, p-value=3.09×10^-2^, or=1.26, 95%CL, 1.02-1.41) and *genus-Phascolarctobacterium* (beta=0.11, se=0.05, p-value=2.63×10^-2^, or=1.12, 95%CL, 1.01-1.23) (**Figure 3B**).

#### 3.2.3 Alzheimer’s disease

We identified four risk factors for Alzheimer’s disease: *order-Desulfovibrionales*(beta=0.20, se=0.10, p-value=3.49×10^-2^, or=1.22, 95%CL, 1.01 = 1.48), *family-Desulfovibrionaceae*(beta=0.20, se=0.10, p-value=3.59×10^-2^, or=1.22, 95%CL, 1.01-1.48), *genus-Dorea*(beta=0.33, se=0.15, p-value=2.35×10^-2^, or=1.40, 95%CL, 1.05-1.86) and *genus-Turicibacter*(beta=0.20, se=0.09, p-value=3.47×10^-2^, or=1.22, 95%CL, 1.01-1.46) (**Figure 4B**).

#### 3.2.4 Brain cancer

Even more surprising is the causal link with brain cancer: *order-Lactobacillales*(beta=1.54×10^-3^, se=5.85×10^-4^, p-value=8.38×10^-3^, or=1.002, 95%CL, 1.0004-1.0027), *family-Oxalobacteraceae*(beta=7.53×10^-4^, se=3.61×10^-4^, p-value=3.69×10^-2^, or=1.0008, 95%CL, 1.00005-1.00146), *genus-Defluviitaleaceae*(beta=-9.98×10^-4^, se=4.58×10^-4^, p-value=2.92×10^-2^, or=0.999, 95%CL, 0.998-0.999), *genus-Escherichia*(beta=-2.12×10^-3^, se=8×10^-4^, p-value=8.05×10^-3^, or=0.998, 95%CL, 0.996-0.999), *genus-Flavonifractor*(beta=-1.44×10^-3^, se=6.93×10^-4^, p-value=3.71×10^-2^, or=0.999, 95%CL, 0.997-0.999) and *genus-Ruminococcus2*(beta=-1.21×10^-3^, se=6.11×10^-4^, p-value=4.74×10^-2^, or=0.999, 95%CL, 0.998-1.000). Although the data suggest a causal link between these GM taxa and brain cancer, the role of GM taxa in promoting or inhibiting the development of brain cancer is not particularly clear (**Figure 5B**).

#### 3.2.5 Parkinson’s disease

We found four very specific protective factors for Parkinson’s disease: *order-Burkholderiales* (beta=-0.59, se=0.29, p-value=3.78×10^-2^, or=0.55, 95%CL, 0.32-0.97), *family-Acidaminococcaceae* (beta=-0.63, se=0.26, p-value=1.58×10^-2^, or=0.535, 95%CL, 0.321-0.889), *genus-Intestinibacter* (beta=-0.37, se=0.18, p-value=4.37×10^-2^, or=0.69, 95%CL, 0.48-0.99) and *genus-Phascolarctobacterium* (beta=-0.58, se=0.20, p-value=3.34×10^-3^, or=0.56, 95%CL, 0.38-0.82) (**Figure 6B**).

#### 3.2.6 Bipolar disorder

Nine clear bipolar disorder cause-and-effect relationships have been established: only *genus- Candidatus* (beta=-0.20, se=0.08, p-value=3.34×10^-3^, or=0.56, 95%CL, 0.38-0.82) was found to have an antagonistic effect against bipolar disorder. The rest, including *class-Bacilli*(beta=0.23, se=0.07, p-value=4.09×10^-3^, or=1.25, 95%CL, 1.07-1.453), *class-Betaproteobacteria*(beta=0.22, se=0.09, p-value=2.16×10^-2^, or=1.24, 95%CL, 1.03-1.50), *order-Burkholderiales*(beta=0.29, se=0.10, p-value=4.56×10^-3^, or=1.33, 95%CL, 1.09-1.63), *order-Lactobacillales* (beta=0.24, se=0.08, p-value=2.55×10^-3^, or=1.27, 95%CL, 1.09-1.49), *family-Alcaligenaceae*(beta=0.27, se=0.11 p-value=1.26×10^-2^, or=1.31, 95%CL, 1.059-1.62), *genus-Butyricimonas*(beta=0.22, se=0.10, p-value=3.05×10^-2^, or=1.25, 95%CL, 1.02-1.54), *genus-Erysipelatoclostridium*(beta=0.14, se=0.07, p-value=4.61×10^-2^, or=0.1.15, 95%CL, 1.00-1.31) and *genus-Roseburia* (beta=0.39, se=0.11, p-value=3.92×10^-4^, or=0.88, 95%CL, 0.77-1.00) are bipolar disorder’s risk factors (**Figure 7B**).

#### 3.2.7 Stroke

There are five protective factors for stroke in all the causal relationships we found: *order-Desulfovibrionales* (beta=-0.14, se=0.07, p-value=3.91×10^-2^, or=0.87, 95%CL, 0.77-0.99), *family-Desulfovibrionaceae* (beta=-0.13, se=0.07, p-value=4.03×10^-2^, or=0.87, 95%CL, 0.77-0.99), *genus-Barnesiella* (beta=-0.13, se=0.06, p-value=4.25×10^-2^, or=0.88, 95%CL, 0.77-1.00), *genus-Candidatus* (beta=-0.15, se=0.07, p-value=3.09×10^-2^, or=0.86, 95%CL, 0.75-0.98) and *genus-Coprococcus3* (beta=-0.20, se=0.08, p-value=1.12×10^-2^, or=0.82, 95%CL, 0.70-0.96). Moreover, *genus-Alistipes* (beta=0.17, se=0.08, p-value=3.93×10^-2^, or=1.19, 95%CL, 1.01-1.40) and *genus-Paraprevotella* (beta=0.08, se=0.04, p-value=3.95×10^-3^, or=1.08, 95%CL, 1.00-1.16) are risk factors for stroke (**Figure 8B**).

### 3.3 Sensitivity analyses

The MR-Egger, weighted mode, simple mode, and weighted median methods yielded similar causal estimates for magnitude and direction. We found no evidence of horizontal pleiotropy for gut microbiota in neurological disorders with p > 0.05 when using the MR-Egger regression intercept approach. MR-PRESSO analysis revealed no outliers in the results. In the absence of heterogeneity and pleiotropy, the results of IVW were trustworthy. (**Table 3**) **Supplementary Table 1-5**-**Supplementary Table 7-5** shows the pleiotropy and heterogeneity test results for all bacterial taxa and GM viewed as a whole.

**Table 3:** Sensitivity analysis between GM and neurological disorders.

Level	Exposure	Outcome	IVW_Q	IVW_Q_P	MR_Egger_Q	MR_Egger_Q_P	egger_intercept	pleiotropy_P
class	Bacilli	Epilepsy	5.919761	0.747921	3.861166	0.869427	0.02894	0.189264
class	Betaproteobacteria	Epilepsy	4.641785	0.590506	3.733442	0.588395	0.031892	0.384323
order	Burkholderiales	Epilepsy	2.660826	0.914508	2.342741	0.885636	0.016573	0.5932
order	Lactobacillales	Epilepsy	7.91426	0.44189	5.702851	0.574842	0.03466	0.180588
family	Streptococcaceae	Epilepsy	6.426552	0.37714	5.257239	0.3853	-0.04049	0.339896
genus	Coprococcus2	Epilepsy	4.467323	0.45683	5.679543	0.988312	0.003291	0.324239
phylum	Firmicutes	Schizophrenia	4.161719	0.526375	2.46737	0.650488	0.023295	0.262933
class	Clostridia	Schizophrenia	10.72851	0.150907	4.435409	0.61797	0.019219	0.04599
order	Clostridiales	Schizophrenia	10.72783	0.15094	4.445309	0.616646	0.019174	0.046121
genus	Bilophila	Schizophrenia	14.27553	0.139514	9.28704	0.054312	0.049227	0.216578
genus	Desulfovibrio	Schizophrenia	4.4775	0.345224	4.427998	0.218803	0.005976	0.866371
genus	Gordonibacter	Schizophrenia	5.566566	0.518017	0.926087	0.954362	-0.000298	0.97876
genus	Marvinbryantia	Schizophrenia	4.575859	0.469798	4.459555	0.34737	0.005505	0.762886
genus	Phascolarctobacterium	Schizophrenia	4.2428	0.515014	4.060188	0.397922	-0.00861	0.693272
order	Desulfovibrionales	Alzheimer's disease	5.656203	0.580416	5.602481	0.469158	0.004284	0.824412
family	Desulfovibrionaceae	Alzheimer's disease	5.704107	0.574692	5.635804	0.465197	0.004799	0.802564
genus	Dorea	Alzheimer's disease	4.605332	0.33024	3.702468	0.295436	-0.03601	0.455249
genus	Turicibacter	Alzheimer's disease	3.390417	0.64003	3.389401	0.494893	-0.00204	0.9761
order	Lactobacillales	brain cancer	9.566566	0.296774	9.038478	0.249905	-8.15E-05	0.542839
family	Oxalobacteraceae	brain cancer	2.791313	0.834546	1.587422	0.902766	2.80E-04	0.322554
genus	Defluviitaleaceae	brain cancer	6.182599	0.403049	5.637984	0.343053	1.32E-04	0.518038
genus	Escherichia	brain cancer	3.510522	0.3194	0.829037	0.660658	-0.00026	0.243174
genus	Flavonifractor	brain cancer	0.898425	0.825808	0.035239	0.982535	2.58E-04	0.45093
genus	Ruminococcus2	brain cancer	3.674101	0.597223	3.503467	0.477351	5.37E-05	0.700732
order	Burkholderiales	Parkinson's disease	12.66797	0.080623	11.56241	0.072474	-0.05197	0.477472
family	Acidaminococcaceae	Parkinson's disease	2.372807	0.498717	1.288843	0.524966	0.064952	0.407139
genus	Intestinibacter	Parkinson's disease	3.730759	0.713057	3.71844	0.590622	-0.00613	0.91594
genus	Phascolarctobacterium	Parkinson's disease	2.569594	0.765979	2.24373	0.691032	-0.04673	0.598645
class	Bacilli	bipolar disorder	3.823265	0.922655	3.608982	0.890569	-0.00676	0.655762
class	Betaproteobacteria	bipolar disorder	4.993947	0.660702	4.941485	0.55134	0.005205	0.826441
order	Burkholderiales	bipolar disorder	8.398746	0.298749	7.326431	0.291711	0.022655	0.384872
order	Lactobacillales	bipolar disorder	3.096693	0.928144	3.032334	0.881995	-0.00418	0.807025
family	Alcaligenaceae	bipolar disorder	7.500598	0.277019	6.681261	0.245445	0.027823	0.469047
genus	Butyricimonas	bipolar disorder	14.05815	0.050157	11.1065	0.08514	0.039443	0.253532
genus	Candidatus	bipolar disorder	1.284184	0.732892	0.424325	0.808833	-0.02855	0.451671
genus	Erysipelatoclostridium	bipolar disorder	2.938008	0.890677	2.178884	0.902533	0.021238	0.417097
genus	Roseburia	bipolar disorder	3.161843	0.675051	3.161369	0.531193	5.71E-04	0.983674
order	Desulfovibrionales	Stroke	9.951202	0.191356	9.759805	0.135138	-0.00472	0.743281
family	Desulfovibrionaceae	Stroke	9.994405	0.188891	9.781436	0.134163	-0.00495	0.730159
genus	Alistipes	Stroke	3.444137	0.486423	2.971643	0.396018	-0.02603	0.541215
genus	Barnesiella	Stroke	12.7169	0.17584	12.03853	0.149493	-0.01068	0.520867
genus	Candidatus	Stroke	4.600963	0.203459	2.943065	0.229573	-0.02902	0.399719
genus	Coprococcus3	Stroke	2.593583	0.62796	0.912757	0.822348	0.022097	0.285532
genus	Paraprevotella	Stroke	5.882194	0.660426	5.879136	0.553931	-8.08E-04	0.957444

## 4 Connection of GM and comparison to other researches

**Figure 9:**
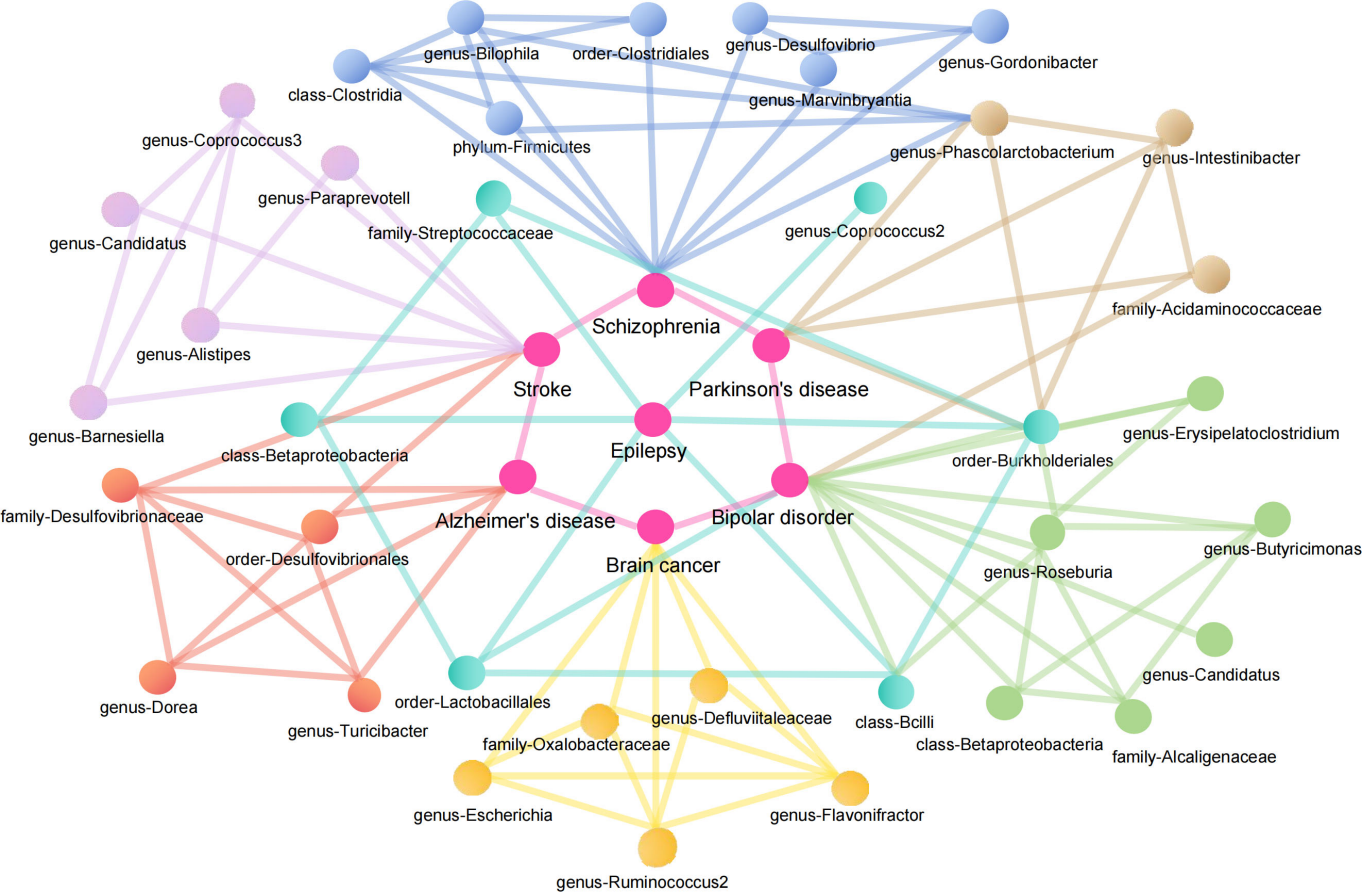
Causal relationship between gut microbiota and neurological diseases and possible links between gut microbiota.

Linking the MR Results of seven neurological diseases, we can obtain a complex network relationship map. It not only shows the taxa of gut microbiota that are strongly associated with each disease, but also shows that one gut microbiota does not necessarily affect only one disease. For example, *genus-Phascolarctobacterium* can be both a protective factor for Parkinson’s disease and a risk factor for Schizophrenia. *Order-Burkholderiales* even has a causal relationship with Parkinson’s disease, Epilepsy and Bipolar disorder. In addition, different gut microbiota taxa were connected by wires to represent their associations. To name a few, *genus-Ruminococcus2* and *genus-Flavonifractor* can promote the decomposition of dietary substances in food (Tian et al., 2021; Yang et al., 2021), while *family-Oxalobacteraceae* can decomcause fiber and other polysaccharides. It helps our body to obtain more nutrients (Hiel et al., 2019; Crivelli et al., 2020). They all play an important role in maintaining the balance of intestinal environment and human nutrition intake. **Figure 9** illustrates the causal relationship between gut microbiota and neurological diseases and possible links between gut microbiota.

Researches studying epilepsy find that *genus-Eubacterium Xylanophilus Group*, *Genus-Unknown genus* can decrease the risk of epilepsy, and that *class-Betaproteobacteria, class-Verrucomicrobiae, order-Burkholderiales, genus-Anaerotruncus* which have many common points to our research (Zeng et al., 2023). Schizophrenia is a complex psychiatric disorder with poorly understood etiology. To date, only few studies have investigated differences in the gut microbiota between patients suffering from schizophrenia and healthy subjects. In most of them, no changes in microbial richness/diversity were reported. There were, however, some marked differences in the abundance of specific taxa between schizophrenic patients and control groups but with much discrepancy between the reports. In the study by He et al, no significant differences in microbial diversity were observed between high-risk patients, ultra-risk patients, and healthy controls. Interestingly, the orders *Clostridiales*, *Lactobacillales*, and *Bacteroidales*, *Andgenera*, *Lactobacillus* and *Prevotella* were significantly increased in the ultra-risk patients as compared to the other two groups (He et al., 2018). In our research, we find that *genus-Desulfovibrio, genus-Gordonibacte, genus-Marvinbryantia* can decrease the risk of schizophrenic, and that *phylum-Firmicutes, class-Clostridia, order-Clostridiales, genus-Bilophila, genus-Phascolarctobacterium* are schizophrenic’s risk factors. Additionally, there are many researches studying Parkinson’s disease, we analyzed the common points in these researches. It is concluded that *phylum-Lentisphaerae, class-Lentisphaeria, genus-Anaerostipes, genus-Bifidobacterium, order-Victivallales* are the protective factors (Ning et al., 2022; Jiang et al., 2023; Ji et al., 2024). As for our study, we also find that *order-Burkholderiales, family-Acidaminococcaceae, genus-Intestinibacter, genus-Phascolarctobacterium* can also be the protective factors. And the risk factors such as *order-Bacillales, family-Oxalobacteraceae, genus-Bifidobacterium, genus-Eubacterium hallii group, genus- Romboutsia* are also gained.

As for the other neurological disorders including Alzheimer’s disease, bipolar disorder, stroke, we can furtherly determine valuable indicators for disease evolution tracking and potential treatment targets through analyzing the similarity and difference between other researches and our study. We find that *order-Bacillales* have an antagonistic effect against Alzheimer’s disease, and *class-Actinobacteri* (Fu et al., 2023; Ji et al., 2024), f*amily-Desulfovibrionaceae, genus-Sellimonas, order-Desulfovibrionales* can increase the risk of Alzheimer’s disease (Ning et al., 2022). With regard to stroke, we extract the same results between researches on stroke. The results show that g*enus-Desulfovibrio, genus-Barnesiella, genus-Coprococcus* can decrease the risk of stroke, and that *genus-Alistipes* can promote stroke (Lin et al., 2023; Wang et al., 2023). Only few studies have investigated the link between bipolar disorder and gut flora. However, it is determined that *class-Betaproteobacteria* can decrease the risk of bipolar disorder (Ni et al., 2021). Apart from that we also detect *order-Burkholderiales, order-Lactobacillales* and so on can also be the risk factors. It is worth mentioning our study is the first MR investigation to investigate the causal link between brain cancer and gut microbiota. So it is necessary to conduct more research to deepen our understanding of connections and causality between brain cancer and gut microbiota (**Table 4**).

**Table 4:** Summary of Mendelian randomization results for gut microbiota and neurological diseases.

Other Studies				Our study		
Disease	PMID	Protective factor	Risk factor	Disease	Protective factor	Risk factor
PD	35275534	phylum-Lentisphaerae	order-Bacillales	PD	order-Burkholderiales	
		class-Lentisphaeria	family-Oxalobacteraceae		family-Acidaminococcaceae	
		order-Victivallales	genus-Eubacteriumhalliigroup		genus-Intestinibacter	
		genus-Anaerostipes	genus-clostridiumsensustricto1		genus-Phascolarctobacterium	
	37159496	genus-Bifidobacterium	genus-Bifidobacterium			
			order-Bacillales			
			genus-Candidatus Soleaferrea			
			genus-Clostridium sensustricto1			
			genus-Eubacterium hallii group			
			genus-LachnospiraceaeUCG010			
			Genus-Senegalimassilia			
	38178103	order -Victivallales	genus- Romboutsia			
		class -Lentisphaeria	genus- Roseburia			
			Genus- Lachnoclostridium			
			class -Erysipelotrichia			
			phylum- Lentisphaerae			
	38287957	genus-Butyricimonas				
		Phylum-Lentisphaerae				
AD	35275534	genus-Faecalibacterium	class-Actinobacteria	AD	order-Desulfovibrionale	
		genus-Ruminiclostridium9	family-Lactobacillaceae		family-Desulfovibrionaceae	
			genus-Lachnoclostridium		genus-Dorea	
			genus-Ruminiclostridium6		genus-Turicibacter	
	38257137		class-Actinobacteria			
	38178103	family-Defluviitaleaceae	genus-Allisonella			
		genus-Anaerotruncus	genus-Lachnospiraceae FCS020 group			
		order-Bacillales	genus-Sellimonas			
	38287957	order-Bacillales	family-Desulfovibrionaceae			
			genus-Sellimonas			
			order-Desulfovibrionales			
Epilepsy	36922970	genus-Eubacterium Xylanophilus Group	class-Betaproteobacteria	Epilepsy	genus-Coprococcus2	class-Bcilli
		genus-Unknown genus	class-Verrucomicrobiae			class-Betaproteobacteria
			order-Burkholderiales			order-Burkholderiales
			order-Verrucomicrobiales			order-Lactobacillales
			family-Verrucomicrobiaceae			family-Streptococcaceae
			genus-Akkermansia			
			genus-Anaerotruncus			
			genus-Ruminococcaceae UCG 014			
Schizophrenia	34841075	Lachnospiraceae	Bacteroidales_S24-7	Schizophrenia	genus-Desulfovibrio	phylum-Firmicutes
		Lactobacillaceae	Prevotellaceae		genus-Gordonibacte	class-Clostridia
		Verrucomicrobiaceae			genus-Marvinbryantia	order-Clostridiales
						genus-Bilophila
						genus-Phascolarctobacterium
Brain cancer	36627748	phylum-Verrucomicrobia		Brain cancer		order-Lactobacillales
		genus-Akkermansia				family-Oxalobacteraceae
		genus-Bifidobacteriu				genus-Defluviitaleaceae
		genus-Actinobacteria				genus-Escherichia
		genus-Firmicutes				genus-Flavonifractor
		genus-Bacteroidetes				genus-Ruminococcus2
Bipolar disorder	35185808		class-Betaproteobacteria	Bipolar disorder	genus-Candidatus	class-Betaproteobacteria
						order-Burkholderiales
						order-Lactobacillales
						family-Alcaligenaceae
						genus-Butyricimonas
						genus-Erysipelatoclostridium
						genus-Roseburia
Stroke	37813672	genus-Tyzzerella3	genus-Blautia	Stroke	order-Desulfovibrionales	genus-Alistipes
		order-Bifidobacteriaceae	genus-Butyricicoccus		family-Desulfovibrionaceae	genus-Paraprevotella
		family-bifidobacteriaceae			genus-Barnesiella	
		genus-Coprococcus1			genus-Candidatus	
		genus-Romboutsia			genus-Coprococcus3	
		genus-Desulfovibrio				
		genus-Clostridiuminnocuumgroup				
		genus-Ruminococcusgauvreauiigroup				
	38029236	Barnesiella	Allisonella			
		Intestinimonas	Paraprevotella			
		LachnospiraceaeFCS020group	Streptococcus			
		LachnospiraceaeNK4A136group				
		RuminococcaceaeUCG004				

PD, Parkinson's disease; AD, Alzheimer's disease.

## 5 Disscussion

Compared to other researches, this is the first MR investigation to thoroughly investigate the causal link between almost all forms of neurological disorders and gut microbiota. In the biggest GWAS of the gut microbiota, robustly related gene variations were discovered. We discovered genetic liability to certain gut bacteria that is causally connected with neurological illness using extensive genomic data from over 450,000 European people. Surprisingly, genetic susceptibility to *genus-Candida* and *genus-Butyricimonas* was shown to be causally linked to Bipolar illness. We also found 87 distinct taxa that might be risk factors or protective factors for neurological disorders. These findings might have consequences for public health strategies focused at lowering the risk of neurological disorders.

GM is made up of microorganisms found in the human digestive system, such as bacteria, viruses, fungus, and archaea. These microbes are crucial for a variety of physiological and metabolic processes, including nutrition digestion and absorption, immune system development, and vitamin production (Heintz-Buschart and Wilmes, 2018). The composition of GM changes according to age, nutrition, lifestyle, and geographical location (Arumugam et al., 2011). There are, however, several bacterial species that are typically present in the gut microbiota of healthy people. *Bacteroidetes*, *Firmicutes*, *Actinobacteria*, *Proteobacteria*, and *Verrucomicrobia* are among them (Structure, function and diversity of the healthy human microbiome, 2012). Dysbiosis of the gut microbiota may result in a variety of disorders in the body, such as intestinal diseases, metabolic diseases, autoimmune diseases, neurological diseases, and so on (Turnbaugh et al., 2006; Round and Mazmanian, 2009).

For decades, researchers have been studying the link between the gastrointestinal (GI) tract and the brain. The “gut-brain axis” refers to the unique connectivity between the GI tract and the central nervous system (CNS), which consists of bidirectional interaction between the two (Stilling et al., 2014; Dinan and Cryan, 2017). Psychological stress and inflammation are frequent pathophysiologic denominators in disorders in which microbiota may have a role. In depression, schizophrenia (Golofast and Vales, 2020), autism spectrum disorder (ASD) (Theoharides et al., 2019), epilepsy (Comerford et al., 1988), and migraine (Goadsby et al., 2017), stress plays a role, whereas inflammation plays a role in depression, schizophrenia (Golofast and Vales, 2020), ASD (Matta et al., 2019), Parkinson’s disease (Rocha et al., 2018), epilepsy (Mazarati et al., 2017), and migraine (Goadsby et al., 2017). Furthermore, the disorders listed above often coexist. Depression and ASD, for example, are prevalent co-morbidities in epilepsy. Migraine and depression often co-exist (Mazarati et al., 2017). Furthermore, there is a greater frequency of gastrointestinal illnesses in migraine sufferers, such as inflammatory bowel disease or irritable bowel syndrome (IBS) (Van Hemert et al., 2014). The vagal nerve, tryptophan metabolites, and microbial products such as short-chain fatty acids (SCFAs) or peptidoglycan are important communication pathways between the intestinal microbiota and the brain (Dinan and Cryan, 2017; Foster et al., 2017). The microbiota in the stomach may influence brain function by altering serotoninergic, noradrenergic, dopaminergic, glutamatergic, and GABA-ergic neurotransmission (Fendt et al., 2008; Winter et al., 2018). Microbiota may either impact neurotransmitter synthesis/metabolism or manufacture these neuroactive chemicals on their own. With the exception of GABA, which is found in the blood-brain barrier (BBB), neurotransmitters generated in the stomach are unlikely to reach the brain due to the existence of the BBB. Neurotransmitters generated in the stomach, on the other hand, may have an indirect effect on the brain by acting on the enteric nervous system (ENS) (De Caro et al., 2019). Furthermore, the gut microbiota contains enzymes that regulate tryptophan metabolic pathways, leading to the production of serotonin, kynurenine, or indole derivatives. Microbiota impact the quantity of serotonin in the brain via changing the amount of serotonin precursor - tryptophan.

The makeup of the gut microbiota is controlled by environmental variables and, to a lesser degree, host genetics (Rothschild et al., 2018). Diet is a key determinant affecting the makeup of the intestinal microbial community (Falony et al., 2016; Makki et al., 2018), and the result of a dietary intervention is impacted by the composition of the gut microbiota at the time of intervention (Griffin et al., 2017). Certain forms of dietary fibers known as microbiota-accessible carbohydrates (MACs) supply a crucial energy source to a healthy intestinal microbiota (Gmeiner, 2014). The ketogenic diet is particularly fiber-deficient, and a few recent studies have explored alterations in the gut microbiota in individuals with epilepsy during KD, which include decreased relative abundance of fiber-consuming bacteria such as *bifidobacterial* (Bordin et al., 2018). In our study, *genus-Coprococcus2*, a cellulose-consuming bacterium, was also a protective factor against epilepsy. This is consistent with the above phenomenon. At the same time, the risk findings of *genus-class-Betaproteobacteria* and *order-Burkholderiales* were highly consistent with previous studies. Safak et al. found the *genus-Delftia* and *genus-Lautropia*, both members of the *family-Burkholderiales*, to be considerably higher in the intestines of epilepsy patients compared to healthy people (Şafak et al., 2020). Furthermore, another genus of *Burkholderiales*, *Sutterella*, has been linked to increased intestinal abundance in adult epileptic patients (Dong et al., 2022). One crucial factor to consider is that the microbiota has a significant role in suppressing the immune system, promoting inflammation, and supporting many cellular processes such as proliferative signaling, cell death limitation, angiogenesis, and invasiveness (Mehrian-Shai et al., 2019). Simultaneously, a substantial body of data supports the notion that a significant proportion of neurological disorders exhibit immune-related characteristics, such as the activation of glial cells, the presence of cytokines, chemokines, and reactive oxygen species. Inflammation, then, emerges as a crucial mechanism underlying neurological disorders. Multiple studies have shown that individuals diagnosed with epilepsy, schizophrenia, Alzheimer’s disease, brain cancer, Parkinson’s disease, Bipolar disorder, and Stroke have diverse levels of inflammation within the neurological system (Vezzani et al., 2011; Heneka et al., 2015; Khandaker et al., 2015; Chamorro et al., 2016; Saccaro et al., 2021; Mundt et al., 2022; Tansey et al., 2022). In the present investigation, a number of bacterial species were identified that are known to be linked to inflammatory processes and immune responses. The bacterium known as *order-Lactobacillus*, belonging to the *bacilli* category, has long been recognized as a probiotic. However, it is noteworthy that it can also serve as a risk factor for Epilepsy and Bipolar disorder. This unexpected association may be attributed to the imbalance of the gut microbiota, known as dysbiosis, and the subsequent alteration of the production of short-chain fatty acids (SCFAs). These changes have the potential to disrupt the communication between the gut and the brain, thereby influencing neuroinflammatory processes. Several studies have shown that the consumption of modest amounts of SCFAs may provide benefits to the nervous system, exhibit anti-inflammatory and antioxidant properties, and may mitigate inflammatory reactions. Nevertheless, in the event of an imbalance in the gut microbiota or other related circumstances, the overproduction of short-chain fatty acids might potentially result in the impairment of the gut barrier (Parada Venegas et al., 2019). This phenomenon might potentially be associated with the presence of inflammatory processes seen in some neurological diseases. Additionally, potential processes that might be considered include the following: *order-Burkholderiales* in relation to epilepsy, *class-Clostridia*, *order-Clostridiales*, *genus-Bilophila*, *genus-Phascolarctobacterium*, *genus-Dorea*, *genus-Butyricimonas*, and *genus-Roseburia*. The gut microbiota often has a favorable association with short-chain fatty acids, which frequently manifest anti-inflammatory properties (Morrison and Preston, 2016). Nevertheless, it is important to note that results may exhibit variability, potentially leading to the promotion of chronic inflammation and the development of illness in some circumstances. The findings of our study suggest a potential association between dysbacteriosis in the gastrointestinal tract and an overabundance of short-chain fatty acids, which may contribute to the development of chronic inflammation within the neurological system (Liu et al., 2021). It is widely acknowledged that certain bacteria, specifically those belonging to the *genus-Coprococcus2*, *genus-Marvinbryantia*, and *genus-Coprococcus3*, as well as the *order-Burkholderiales*, play a significant role in the process of fermenting dietary fiber. This fermentation process leads to the production of short-chain fatty acids, which serve to safeguard the integrity of the intestinal mucosa and mitigate the impact of inflammation (Deleu et al., 2021). Consequently, these bacteria contribute to the deceleration of neurological disorders, such as Parkinson’s disease. A more innovative finding was the theoretical identification of six taxas associated with brain cancer: *order-Lactobacillales*, *family-Oxalobacteraceae*, *genus-Defluviitaleaceae*, *genus-Escherichia*, *genus-Flavonifractor*, *genus-Ruminococcus2*. Unfortunately, the beta and or values did not show a significant difference, which means that we cannot infer from this the specific role of these flora in the progression of brain cancer. But a comparison with previous research suggests something: *order-lactobacillales* and *genus-Ruminococcus2* may play a role in anti-inflammation (Vemuri et al., 2017; Fang et al., 2023), and there is abundant evidence that brain cancer development is associated with immune system disorders, angiogenesis, and inflammation (Kleffman et al., 2022). Taken together, it’s not hard to see how the gut microbiota can have a big impact on neurological disorder, and how inflammation and the immune system can act as a bridge between the two.

This research provides a number of noteworthy advantages. First, our analysis takes a more detailed approach by looking at the causal influence of each GM taxon on neurological disorders from the genus to the phylum level, in contrast to earlier studies on the connection between GM and neurological disorders that have primarily focused on family-level classification. This method gives a conceptual framework for examining the roles that certain bacterial strains play in neurological disorders and yields a plethora of insightful clinical data, such as the rise in Firmicutes, which was previously thought to be connected to obesity (Yee et al., 2019). This could be a targeted therapy meant to lower the prevalence of obesity-related schizophrenia. Second, our results are more credible than those of smaller randomized controlled trials because we have analyzed genetic data from a large sample size by using the most recent large-scale genome-wide association studies. Moreover, Mendelian randomization analysis clears up any ambiguity and offers a new angle on investigating the workings of the “gut-brain axis.”

By contrast, there are some differences between our results and those of other MR studies related to neurological diseases. In general, for the same exposure and outcome in MR, differences in analytical results arise from several factors, including data quality, selection of instrumental variables, statistical methods, pleiotropy issues, sample size and representativeness, and random error. Data quality and processing methods can directly affect the reliability of the results, including data loss and outlier handling, as well as errors in the preprocessing steps. Secondly, differences in the genetic variants selected as exposure(instrumental variables) can also have a significant impact on the results, and different genetic variants may have different effects on the exposure factors, leading to differences in the results. Of course, the method selection of MR will also lead to different results, such as the selection of inverse variance weighting method or weighted median method, which will lead to different analysis results. As an example, although this study of ours fulfilled the three major assumptions of MR Analysis, as did other MR studies focusing on GM, and the IVs used were closely related to gut microbiota taxa, there would still be the possibility of some tool bias. To determine the credibility and reliability of the studies, both our study and Zeng’s study applied a series of tests, including inverse variance weighting, weighted mean, weighted median, and MR-Egger test. However, Zeng also added simple mode in it. In addition, the Bonferroni method was used for multiple testing correction in their study. The threshold for various level is p < 0.05/n, where n represents the number of taxa at a particular level (Morton et al., 1987). In addition, pleiotropy of the IVs selected by MR, which affects both the exposure factor and other factors related to the outcome, may also bias the results. Whereas, testing for pleiotropy in our study ruled out this source of error. It is worth mentioning that the size and representativeness of the sample can also influence the final results. Differences in the number of samples or representativeness of the samples for the outcomes obtained in different studies may lead to variations in the results. Our respective database selections for exposure were not necessarily the same as those in the studies of others, so it is plausible that our analyses will differ. Moreover, all statistical analyses are subject to random error, and even if all other conditions are equal, it may be due to random error that results in different analyses differ. Therefore, a series of sensitivity analyses, such as confounding adjustment, outlier treatment, comparison of results between different methods, single point deletion analysis, overidentification test and Steiger directionality, are needed to ensure the stability and reliability of the results.

Of course, some of our results are the same as those of others, which means that we have again validated the relationship between gut microbiota and a variety of neurological diseases in different samples, strengthening the strength of evidence for this relationship. For example, in our MR Analysis of Alzheimer’s disease, Two taxa order-Desulfovibrionale and family-Desulfovibrionaceae that we identified as risk factors were also confirmed as risk factors in another study on gut microbiota and dementia (Fu et al., 2023). At the same time, our findings of two taxa class-Betaproteobacteria and order-Burkholderiales, risk factors for epilepsy, were consistent with the results of Zeng et al. ‘s study, even though the MR Methods used in our two studies were not exactly the same (Zeng et al., 2023). In addition, in an article comparing the relative abundance of gut microbiota at different levels between brain cancer patients and healthy patients, genus-Escherichia was found to be a biomarker of malignant brain tumors and has been shown to be associated with cancer, which may be a potential risk for brain cancer development. In our study, genus-Escherichia was confirmed as a risk factor for brain tumors (Lin et al., 2023). It is worth noting that in the study on BD, Ni et al. separately investigated the relationship between one taxon, class-Betaproteobacteria, and BD, and finally found it to be a risk factor for BD (Ni et al., 2021). Similarly, in our MR Results, IVW showed that class-Betaproteobacteria was one of the risk factors for BD. The consistency between the results of these studies not only makes the robustness of our study conclusion more convincing, which is not limited by specific samples or populations, but also reflects the scientific validity and effectiveness of our study design and analysis method, which increases the practical value of the study.

From the research we have done, it is undeniable that our study has many limitations due to the cohort from the FinnGen programme that was employed for the examination of neurological illnesses. Essentially, this means that the patients in the research who were included had enough control over their age and other variables, and that quality monitoring that was done to guarantee the correctness of the diagnosis was absent. Building on this work, we want to carry out more research in the future. We want to use information from many centers. To make this research more thorough, we will duplicate our results using the neurological disorders cohort from the sizable International neurological disorders Genetics collaboration and combine it with the data from our own gathered cases. Furthermore, using a number of statistical modifications might be too rigorous and conservative, leaving out GM taxa that may be causally related to neurological diseases. Therefore, we skipped over looking at the outcomes of further testing given the biological plausibility of our findings. We were unable to show a causal association between neurological illnesses and any specific GM species, despite being the first research to apply MR analysis to evaluate the relationship between GM taxa and neurological disorders risk at the species level. Further research examining the relationship between GM taxa and neurological problems at the species level, with a larger sample size, is necessary to provide further theoretical support for the investigation of the “gut-brain” axis mechanism.

By summarizing emerging data on the role of “gut-brain” axis in the pathophysiology of neuropsychiatric and neurological disorders, our review presents a comprehensive overview of the potential involvement of the human gut microbiota in the pathogenesis of neurological disorders. Not only based on our own empirical research, but also actively refer to and summarize the results of other researchers in the same major. In most cases, our research results echo the research findings of others and verify each other, which undoubtedly strengthens the reliability and objectivity of our research topics. At the same time, our research has also found some new causal relationships between them, and these conclusions provide new insights for us to understand the complex interaction between intestinal flora and neurological diseases. For instance, our work has demonstrated a causal relationship between the risk of neurological disorders development and particular GM taxa, such as the *order-Lactobacillales*, *family-Oxalobacteraceae*, *genus-Defluviitaleaceae*, *genus-Escherichia*, *genus-Flavonifractor*. Our results imply that these GM taxa may provide new opportunities for the development of neurological disorders treatments and preventative measures, as well as potential biomarkers.
